# Nonstenotic Culprit Plaque: The Utility of High-Resolution Vessel Wall MRI of Intracranial Vessels after Ischemic Stroke

**DOI:** 10.1155/2015/356582

**Published:** 2015-08-06

**Authors:** Adam de Havenon, Chun Yuan, David Tirschwell, Thomas Hatsukami, Yoshimi Anzai, Kyra Becker, Ali Sultan-Qurraie, Mahmud Mossa-Basha

**Affiliations:** ^1^Department of Neurology, University of Utah, 175 North Medical Drive, Salt Lake City, UT 84132, USA; ^2^Department of Radiology, University of Washington, 325 9th Avenue, Seattle, WA 98104, USA; ^3^Department of Neurology, University of Washington, 325 9th Avenue, Seattle, WA 98104, USA

## Abstract

Intracranial atherosclerotic disease (ICAD) accounts for 9–15% of ischemic stroke in the United States. Although highly stenotic ICAD accounts for most of the strokes, it is assumed that nonstenotic ICAD (nICAD) can result in stroke, despite being missed on standard luminal imaging modalities. We describe a patient with nICAD who suffered recurrent thromboembolic stroke and TIA but had a negative conventional stroke workup. As a result, they were referred for high-resolution magnetic resonance imaging (HR-MRI) of the arterial vessel wall, which identified a nonstenotic plaque with multiple high-risk features, identifying it as the etiology of the patient's thromboembolic events. The diagnosis resulted in a transition from anticoagulation to antiplatelet therapy, after which the patient's clinical events resolved. HR-MRI is an imaging technique that has the potential to guide medical management for patients with ischemic stroke, particularly in cryptogenic stroke.

## 1. Introduction

Intracranial atherosclerotic disease (ICAD) accounts for 9–15% of ischemic stroke in the United States, approximately 70,000 cases yearly, with even higher incidence in Asian, African American, and Hispanic populations [1–3]. With the development of high-resolution magnetic resonance imaging (HR-MRI) of the arterial vessel wall it has become apparent that nonstenotic ICAD (nICAD) can result in stroke [4–6] despite being missed on standard luminal imaging modalities, which may depict a symptomatic plaque as a normal vessel due to the predilection of lesions to outwardly remodel [7, 8]. Several authors have described deep infarcts arising from nICAD of the MCA, likely secondary to lenticulostriate branch ostial occlusion. The features of high-risk extracranial carotid atherosclerosis include intraplaque hemorrhage, thinning/rupture of the fibrous cap, and prominent lipid-rich necrotic core [9]. These risk factors are less well established for ICAD but are presumably similar and hold promise as prognostic indicators of future or recurrent stroke.

Even after a complete diagnostic evaluation, 30–40% of ischemic stroke remains cryptogenic [10]. ICAD with overlying thrombus is a common finding on autopsy after fatal stroke [11], suggesting that this may represent an underappreciated etiology of cryptogenic stroke. To our knowledge, we report the first case of symptomatic nICAD from thrombus attributable to plaque rupture seen on HR-MRI.

## 2. Case Report

**Figure 1:**
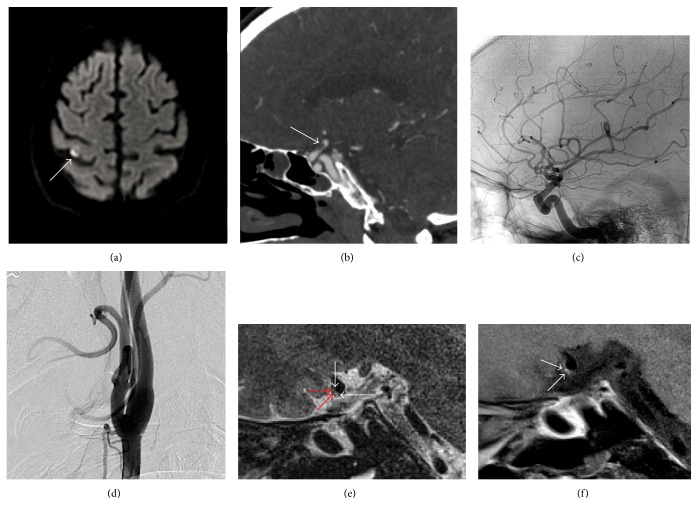
(a) Axial diffusion-weighted MRI showed punctate cortical areas of diffusion restriction (white arrow), consistent with an embolic source of ischemia. (b) Sagittal CT angiogram shows high-grade narrowing (white arrow) of the right supraclinoid ICA, presumed secondary to thrombus which was in close proximity to focal ICA calcification (not seen). (c) Lateral DSA performed two weeks after the prior CTA shows no significant stenosis or other vascular abnormality of the right anterior circulation. (d) Lateral DSA shows a patent right cervical internal carotid artery. (e) Sagittal T2-weighted HR-MRI of the supraclinoid ICA shows atherosclerotic plaque along the anterior wall of the supraclinoid ICA, with discontinuous juxtaluminal T2 hyperintense band (white arrows), and deeper T2 hypointensity representing the lipid necrotic core (red arrows). (f) Sagittal T1 postcontrast HR-MRI at the same level shows T1 hypointense plaque with focal enhancement along the juxtaluminal surface, which was not present on the precontrast T1 image, and a normal to eccentrically enlarged vessel lumen (white arrow).

We describe a middle-aged patient with no relevant past medical history who presented to an outside hospital with a two-day history of left upper extremity paresthesias and weakness. MRI of the brain showed several punctate areas of cortical diffusion restriction around the central sulcus (in the right middle cerebral artery territory) consistent with embolic infarcts (Figure 1(a)). MRA of the head and neck, including the aortic arch, carotid ultrasound, and transesophageal echocardiogram were unremarkable. The patient was started on low-dose aspirin and a statin. After discharge the symptoms improved, but two weeks later the patient had recurrence of left upper extremity paresthesias and weakness with extension of the sensory change into the left leg. The symptoms quickly resolved and repeat MRI showed no new infarcts. Noncontrast head CT showed a focal calcification involving the right supraclinoid ICA. Luminal imaging with CT angiogram showed marked stenosis of the right supraclinoid ICA adjacent to the calcification seen on noncontrast CT (Figure 1(b)), which had resolved on follow-up noncontrast 3D time of flight MR angiogram done later that day. This transient stenosis was thought to be related to a thrombus that resolved. The patient was anticoagulated with intravenous heparin and transitioned to warfarin. They had no identifiable thrombophilia and extended ambulatory cardiac monitoring was unremarkable.

The patient started having daily episodes of worsening of the left-sided symptoms and two weeks later they had another MRI and MRA that was unchanged from prior scans. They were empirically switched from warfarin to clopidogrel. A digital subtraction angiogram (DSA) was performed and showed no stenosis or irregularity of the intracranial or carotid arteries (Figures 1(c) and 1(d)). After discharge the patient was seen in our interventional neuroradiology clinic where physical exam revealed persistent mild left upper extremity weakness and sensory loss but no further fluctuations in subjective symptoms. Given the cryptogenic nature of the stroke, they were referred for HR-MRI of the extracranial carotids and intracranial vasculature. Scans were performed on a Siemens Trio 3T MRI scanner (Siemens Healthcare, Erlangen, Germany) using the parameters seen in Table 1. Extracranial carotid vessel wall imaging of the right carotid bulb showed no evidence of appreciable atherosclerotic plaque (not shown). On intracranial vessel wall HR-MRI, there was nICAD of the right supraclinoid ICA with focal postcontrast enhancement along the juxtaluminal surface of the plaque (Figure 1(f)). There was discontinuous juxtaluminal linear T2 hyperintensity suggestive of fibrous cap thinning (Figure 1(e)) resulting in a high-risk atherosclerotic lesion. Fortunately, the patient remains clinically stable on clopidogrel and statin therapy.

## 3. Discussion

Our patient suffered a multifocal embolic stroke in the territory of the right MCA and subsequent episodes of stereotyped neurologic deficits that were MRI negative but likely represented TIA. The patient had an extensive unremarkable workup for the cause of stroke and, ultimately, HR-MRI revealed nICAD of the right supraclinoid ICA with focal postcontrast enhancement and a discontinuous juxtaluminal T2 hyperintense band. As a diagnosis of exclusion, the high-risk intracranial lesion seen on HR-MRI was interpreted as the most likely etiology of the thromboembolic events. Interestingly, the plaque showed no evidence of luminal stenosis or appreciable irregularity on DSA or MRA.

Multiple authors have reported case series of patients with ischemic stroke attributed to nICAD, which can cause infarcts from occlusion or stenosis at the origin of perforating arteries [4–6]. The largest studies of ICAD show a correlation between increasing stenosis and stroke risk, but these studies did not enroll patients with nICAD and did not characterize morphological characteristics with HR-MRI [12]. Postmortem histological studies of ICAD support the theory that, in addition to stenosis, plaque features such as lipid content and neovascularization are equally important in determining the risk of stroke [13]. While the negative results of the SAMMPRIS trial have dampened enthusiasm for endovascular stenting of ICAD, the low event rate on dual antiplatelet treatment in the medical arm of that trial suggested that a 3-month course of aspirin and clopidogrel after TIA or stroke in ICAD lowers the risk of early stroke recurrence [14]. The recently published CHANCE trial also supports the use of dual antiplatelet therapy in the early period after noncardioembolic stroke in an Asian population, who are statistically more likely to have ICAD [15]. HR-MRI is a promising technique to identify high-risk nICAD that would be missed in a conventional stroke workup, which was the case in our patient and, as a result, can change patient management.

## 4. Conclusion

We describe a patient with nICAD that resulted in thromboembolic ischemic stroke and TIA. CTA initially suggested that a high-grade intracranial stenosis of the right ICA was the etiology, but follow-up luminal imaging of the right ICA showed no evidence of stenosis, raising concern for a resolved thrombus. The source of thrombus was not readily apparent despite extensive workup, leading to diagnostic and therapeutic uncertainty. It was not until we obtained HR-MRI that a nonstenotic plaque with high-risk features became apparent as the culprit. After the resolution of the thrombus, our patient began having TIAs while on warfarin, which stopped once they were switched to clopidogrel. If HR-MRI was done sooner, we would have placed the patient on dual antiplatelet therapy after resolution of the thrombus, based on the results from the SAMMPRIS and CHANCE trials. The technical innovation to perform HR-MRI has gradually become available at larger academic institutions and has the potential to guide medical management for patients with ischemic stroke, particularly in those with cryptogenic stroke. HR-MRI may also have a role in identifying patients that would benefit from endovascular stenting in ICAD. Larger studies are warranted to validate the utility of this promising technology.

## Figures and Tables

**Table 1:** HR-MRI sequences and parameters seen in Figures 1(e) and 1(f) at 3.0 T.

Parameters	2D T2W FSE	2D T1W FSE
TE (ms)	72	10
TR (ms)	3550	1000
FOV (cm)	18 × 18	18.5 × 15.8
Matrix	448 × 448	448 × 448
Slice thickness (mm)	1	2

NA	3	4
Acquisition time per slice (seconds)	10.4	45
Bandwidth	223	207
